# Optimising preoperative expectations to reduce postoperative pain and disability in total hip arthroplasty: a study protocol for a randomised controlled trial

**DOI:** 10.1136/bmjopen-2025-108899

**Published:** 2025-11-13

**Authors:** Ana Ocokoljic, Julia Stuhlreyer, Lena Paschke-Dahl, Sigrid Elsenbruch, Tim Rolvien, Frank Timo Beil, Christian Zöllner, Regine Klinger

**Affiliations:** 1 Department of Trauma and Orthopedic Surgery, University Medical Center Hamburg-Eppendorf, Hamburg, Germany; 2 Department of Anesthesiology, University Medical Center Hamburg-Eppendorf, Hamburg, Germany; 3 Department of Neurology, University Duisburg Essen, Duisburg-Essen, Germany; 4 Helios ENDO-Clinic Hamburg, Hamburg, Germany

**Keywords:** Orthopaedic & trauma surgery, PAIN MANAGEMENT, Patient Participation, Patient-Centered Care, Hip

## Abstract

**Introduction:**

Osteoarthritis (OA) commonly affects the ageing population, particularly the hip joint. Total hip arthroplasty (THA) is a frequent procedure that relieves pain and improves mobility, though some patients experience persistent postoperative pain. With rising numbers of THA, optimising perioperative care and pain management is crucial to address the growing clinical burden and improve patient outcomes. Positive treatment expectations have shown promise in enhancing outcomes, especially in pain management. This study implements two strategies to optimise the patient’s treatment expectations, comprising enhanced physician communication and digital social observational learning. We will examine their separate and combined effects on preoperative expectations, negative emotions, postoperative pain, inflammation and function during recovery up to 12 months postoperatively.

**Methods and analysis:**

This randomised controlled trial (RCT) investigates the impact of augmented physician communication and observational learning on treatment expectations and recovery. Participants (n=200) will be randomised into four groups: treatment as usual (TAU), augmented doctor conversation (aDOC), observational learning video (Video) and a combination of both (aDOC+Video). The aDOC group receives empathic communication and targeted information to strengthen self-efficacy. The Video group watches a model patient demonstrating successful recovery. The combined group receives both interventions. Outcomes will be assessed at multiple time points (4 days preoperatively; 1 and 4 days, 4 weeks and 3, 6 and 12 months postoperatively), including subjective pain ratings, mobility and objective physical function. The primary analysis will compare changes in pain intensity across groups. Secondary outcomes will include functional status, self-efficacy, recovery and systemic inflammatory markers. Statistical analysis involves repeated measures ANOVA and post hoc tests for between-group and within-group comparisons.

**Trial registration number:**

German Clinical Trials Register: DRKS00033212.

**STRENGTHS AND LIMITATIONS OF THIS STUDY**

- This study uses a 2×2 factorial randomised controlled design with four intervention arms.
- The interventions are delivered at two timepoints: 2 days before and 1 day after surgery.
- The sample size was determined by a priori power analysis and accounts for dropouts.
- The subjective and objective outcome measures are assessed longitudinally over 12 months.
- Blinded outcome assessment and standardised randomisation procedures are implemented to minimise bias.

## Introduction

### Background

 Osteoarthritis (OA) is a common condition, especially in the ageing population.^1^ The hip joint is often affected, resulting in severe pain and functional impairments. Total hip arthroplasty (THA) is one of the most frequently performed surgical procedures worldwide, particularly in industrialised countries, such as Germany, providing significant pain relief and improved mobility.^2 3^ THA provides significant short-term and long-term benefits to patients' quality of life, including pain relief, improved functionality and increased overall satisfaction.^4 5^ Despite these benefits, a relevant proportion of patients continues to experience persistent postoperative pain, which affects functional recovery and satisfaction with surgery.^6 7^ As OA incidence continues to rise, preventing postoperative pain, optimising perioperative care and improving pain management remain a major focus of orthopaedic research.^3^

### Optimising treatment expectations to improve acute postoperative pain and prevent chronic pain after surgery

The severity and chronicity of pain following orthopaedic surgery are closely linked to psychological factors, such as preoperative expectations and emotional states.^8 9^ Early efforts to incorporate interventions targeting these expectations in surgical settings have demonstrated positive effects on postoperative pain outcomes, particularly in non-orthopaedic surgeries.^8^,^13^ However, empirical evidence within orthopaedic procedures, including THA, remains limited.

A promising approach in this regard is to target patients’ treatment expectations. Positive expectations have been shown to enhance treatment outcomes and play a crucial role in generating placebo effects, while negative expectations can weaken these effects, contributing to the nocebo phenomena.^14^,^17^

For instance, research has demonstrated that the anticipation of pain can profoundly alter its perception. The expectation of pain can transform a non-painful sensation into a painful experience or, conversely, reduce or even eliminate pain entirely.^18^ In contrast, experimental studies suggest that positive expectations not only modulate pain perception but also influence neural processing, thereby enhancing responses to both placebo and active treatments.^19^ This highlights the significant role of expectations in shaping postoperative pain outcomes, particularly in orthopaedic procedures. Therefore, systematically understanding the mechanisms and effects of expectations may be crucial for improving the prevention of pain and pain treatment efficacy in clinical populations, including patients undergoing THA. By optimising expectations within a therapeutic context, we could potentially enhance recovery, reduce postoperative pain and disability, and prevent chronic pain in a safe and cost-effective manner.^20 21^

### Methods to optimise treatment expectation: observational learning and augmented communication

Treatment expectations are shaped through various mechanisms, including conditioning based on prior experiences.^22^,^24^ Moreover, research suggests that these expectations can be influenced by observing others who have experienced positive treatment outcomes.^1025^,^27^ An additional mechanism relates to communication. Patients often benefit significantly from direct interaction with others, especially healthcare providers. However, such opportunities, for example, augmented communication, are often limited in clinical settings.^28^ While most of the existing evidence on the impact of expectations stems from experimental studies with healthy volunteers, it is important to recognise that the desire for pain relief may differ significantly between healthy individuals and patients undergoing surgery.^24^ In a clinical study, Stuhlreyer *et al*^29^ demonstrated that augmented physician communication aimed at improving treatment expectations significantly reduced postoperative pain after total knee arthroplasty. Building on these findings, it is obvious to address both methods to optimise patients’ treatment expectation and to investigate whether (1.) prerecorded videos of patients who have successfully benefited from the treatment, (2.) physician communication reflecting warmth and competence and (3.) a combination of both can maximise patients’ positive treatment expectations and enhance treatment outcomes.

### Study aims and questions

The aim of this study is to improve postoperative outcomes, particularly pain and functional disability, within the clinical context of pain management in patients undergoing THA. We will investigate the effects of physician communication and social observational learning on the dynamic changes in patients' treatment expectations and, consequently, on postoperative pain perception and functional impairments in patients undergoing THA.

The following are the key questions in this study:

- Can augmented practitioner communication and/or a digital social observation learning intervention effectively optimise preoperative treatment expectations and emotional states in patients undergoing THA?
- Can preoperative expectation management, especially the combination of both strategies, result in improved postoperative health outcomes, especially in terms of pain and functional capacity?
- Do the effects of enhanced communication and social observational learning on expectations and on postoperative subjective and objective outcome dynamics combine in an additive or in a synergistic manner?
- Does expectation management enhance pain-related self-efficacy expectations, reduce preoperative anxiety and stress levels and impact postoperative recovery on subjective and physiological levels, including inflammatory markers?

## Method and Design

### Setting

The study will be conducted at the University Medical Center Hamburg-Eppendorf (UKE), Germany, as part of the Collaborative Research Centre (CRC) SFB 289. In addition to its own study-specific outcomes, the trial also contributes to centrally coordinated assessments defined by the CRC. These centrally collected measures serve overarching research aims and are implemented uniformly across all participating sub-projects (for a preregistration, see: OSF Registries | SFB289 - Central Project NeuroImaging). These centrally coordinated assessments include neuroimaging to examine structural and functional brain connectivity prior to the experimental procedures, as well as the collection of salivary biomarkers such as cortisol awakening response and alpha-amylase activity. In addition, participants complete a standardised set of psychological questionnaires. These data will contribute to cross-project analyses aimed at identifying individual predictors of expectancy-related effects, particularly in relation to stress responsiveness, affective disposition and pain sensitivity.^30^ The study is expected to begin in July 2025, with patient recruitment due to conclude by July 2026. The final follow-up assessments will end in July 2027.

### Study design

We will conduct a RCT using a 2×2 factorial design (physician communication x social observational learning, both with the levels yes/no) with repeated measurements. Patient visits and the administration of questionnaires will be done at various time points: preoperatively, 4 days before surgery (baseline), postoperatively during the hospital stay on day 1 and day 4 (discharge day), as well as at 4 weeks, 3 months, 6 months and 12 months after surgery. In addition to the intervention, patients will be provided with a pain diary and questionnaires to fill out throughout the postoperative period. The follow-up assessments are scheduled at 4 weeks, 3 months, 6 months and 12 months after THA.

The effects of both interventions will be tested on subjective and objective pain measures, including visceral and somatic pain reports (cf. in detail 3).

### Patients

We will include 200 patients undergoing THA at the University Medical Center Hamburg-Eppendorf, UKE, Department of Trauma and Orthopaedic Surgery, performed at a UKE external location (Helios ENDO-Clinic Hamburg).

Participants will be eligible or ineligible based on the following inclusion and exclusion criteria:

#### Inclusion criteria

- Age ≥18 years.
- THA due to OA (diagnosed according to the S3 guidelines of the German Society for Orthopaedics and Trauma Surgery (DGOU)).
- Fluent proficiency in the written and spoken German language.

#### Exclusion criteria

- Presence of one or more malignancies or similar diseases that require treatment.
- Severe acute or chronic mental health condition (e.g., schizophrenia).
- Severe cognitive impairments or dementia.
- Substance abuse (including harmful use of opioids, ICD-10 F11.1, or opioid dependence syndrome, ICD-10 F11.2); note: patients will not be excluded for temporary opioid intake prescribed to bridge the time interval until THA.
- Secondary THA (revision or prior contralateral THA)
- Pregnancy or breastfeeding.

#### Admission and Enrolment of the patients

The preoperative consultation is performed by the treating physician at the Helios ENDO-Clinic in Hamburg, a leading high-volume arthroplasty centre with high expertise. Based on the patient’s symptoms, imaging and established THA guidelines, surgery will be recommended as the appropriate treatment. Following the diagnosis of OA and the planning of primary THA, the practitioner will screen the patient for study eligibility using a checklist. Patient meeting the inclusion criteria will receive detailed information about the study. If they consent to participate, they will be enrolled and subsequently randomised to their respective intervention groups. All participants will receive oral and written information and must provide written informed consent before enrolment. A sample of the patient information and consent form is available in the online supplemental materials.

### Intervention and study groups

The intervention is delivered at two time points: 4 days before surgery (during the preoperative consultation) and on the first postoperative day. These timings are chosen to optimally shape and reinforce patient expectations.

#### Augmented communication-based expectation manipulation (aDOC)

The intervention consists of a structured physician-patient conversation developed for this study, drawing on findings from placebo research and expectation-based interventions in pain and surgical outcomes. It targets both the cognitive dimension, by providing clear and understandable medical information about the procedure, postoperative course and expected recovery, and the emotional dimension, by expressing empathy, warmth, and reassurance.

Randomisation takes place at the baseline visit, approximately 4 days before surgery. On the same day, participants receive their assigned intervention. The physician conversation is during the preoperative visit and focuses on establishing realistic yet positive expectations, addressing potential patients concerns and supporting the patient’s sense of control. The postoperative session, on the first day after surgery, reinforces these expectations, encourages adaptive coping strategies and strengthens the therapeutic relationship.

The intervention is conceptually based on evidence demonstrating that the doctor-patient interaction itself can influence clinical outcomes. Previous studies have shown that positive expectations can reduce symptom intensity and analgesic use, and that the combination of physician warmth and competence produces the strongest effects on patient experience and symptom relief.^8 10 30 31^ The standardised approach ensures consistency while allowing flexibility to respond to individual patient needs.

#### Video-based treatment expectation manipulation

Patients in two of the four groups will watch a 10-min. video containing standardised study information. The video provides insight into another patient’s experience with hip-related pain, functional impairments and the subsequent surgery.

**Figure 1:**
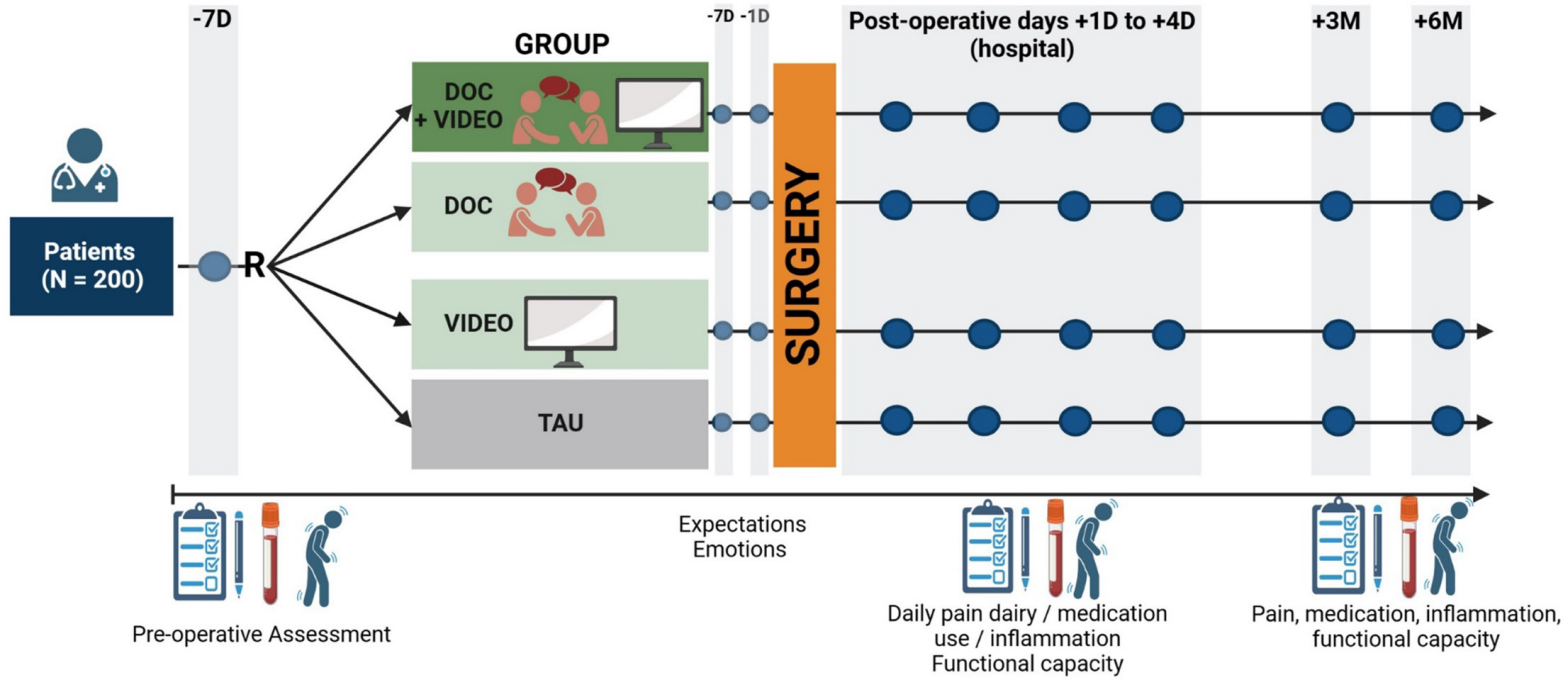
Study design. Schematic illustration of the study design for evaluating the impact of preoperative education on postoperative outcomes in patients undergoing total hip arthroplasty (n=200). Patients are randomised (R) into one of four groups: combined doctor consultation and video intervention (aDOC+VIDEO), doctor consultation only (aDOC), video only (VIDEO) or treatment as usual (TAU). All groups undergo surgery followed by standardised postoperative hospital care. Assessments are conducted at preoperative baseline, daily during the postoperative hospital phase (including pain diary and medication use) and at follow-up visits at 3 months (3M) and 6 months (6M) post-surgery.

In the first part, the clinic director welcomes the viewers and explains the course of their hospital stay. The next section features a patient demonstrating how hip pain and functional limitations affect various everyday activities. This is followed by a depiction of the same activities after the hip arthroplasty, but this time without pain or impairments, illustrating the potential impact of treatment. While the video content is identical, we prepared two different videos to account for potential gender effects, featuring both a male and a female patient. The patients featured in the video are approximately 60 years old, aligning with the target study population.^32^ Participants will be randomly assigned to watch one of the two versions. Only participants of groups 1 and 3 will be shown the video intervention (figure 1). The video is presented twice: first during the preoperative baseline visit and again on the first postoperative day to reinforce what they have learnt and to support expectation maintenance during early recovery.

#### Study groups

After meeting the eligibility criteria and providing written informed consent, participants will be randomly assigned to one of the four study groups.

##### Group 1: aDOC + Video

Participants receive the augmented physician-patient communication during the preoperative and postoperative consultation, in addition to watching a 10-min video depicting a patient who has undergone THA.

##### Group 2: aDOC

Participants receive the augmented physician-patient communication during the preoperative and postoperative consultation, but do not watch the video.

##### Group 3: Video

Participants receive standard clinical consultation but watch the same video as Group 1.

##### Group 4: Treatment as Usual

Participants receive the standard preoperative consultation provided by the clinic, without any additional interventions such as the enhanced conversation or video.

### Blinding and randomisation

The study researcher responsible for analysing the primary outcomes will remain blinded to group allocation by working with a concealed group variable (group variable with hidden label). Since the intervention involves direct delivery by the study physician, the physician must be aware of group assignment to conduct the informed consent process using augmented communication where applicable. To minimise researcher bias, only trained study physicians deliver the augmented consultations (aDOC), while standard consultations (TAU) are provided by clinicians who are not involved in the intervention. Study staff administer the video separately after the physician’s visit, ensuring that consulting physicians remain unaware of whether a participant has received it. This design intentionally introduces a potential experimenter effect, allowing for the investigation of the impact of enhanced physician-patient interaction as part of the intervention itself (‘effects of augmented communication’). Blinding of the study researcher, who is unaware of the group allocation, is designed to minimise this risk as much as possible.

Participants will be randomly assigned to one of the four study groups using a block randomisation method to ensure balanced group sizes throughout the recruitment period. The block size will be kept constant at 20 and will not be disclosed to the physicians involved in recruitment. Allocation concealment will be achieved by using sealed, opaque and sequentially numbered envelopes. Each patient will draw one envelope on inclusion, thereby determining their group assignment by chance. This procedure ensures both randomisation integrity and transparency while minimising selection bias.

## Objectives and Outcomes

### Primary objective (subjective pain measure)

The primary objective of this study is to reduce postoperative pain by optimising preoperative treatment expectations. Pain will be assessed using a numerical rating scale (NRS 0–10) documented in a patient diary, and the Pain and State of Health Inventory (PHI). The PHI captures both pain intensity and the subjective experience over time, enabling a multidimensional evaluation of postoperative pain.

### Secondary objectives

#### Subjective outcome measures

The secondary objectives include the reduction of postoperative functional impairments and evaluating the overall pain area, including its localisation and spatial extent, by distinguishing between interoceptive pain (bone/hip area) and exteroceptive pain (wound/surface).^33 34^

In addition, the study will investigate dynamic changes in the formation of patients’ expectations over time. Furthermore, we aim to clarify the role of self-efficacy in shaping these expectations both before and after surgery. Expectations will be analysed in terms of both an outcome (change over time) and potential moderation of the treatment effects on postoperative outcomes, such as pain and functional recovery.

The functional status and pain related to hip conditions will be assessed with the Harris Hip Score.^35^ The Quality of Recovery-15 questionnaire^36^ will be used to measure the quality of recovery, while the PHI^37^ will be used to quantify the impact of negative mood and distress on quality of life during the acute postoperative period. Self-efficacy will be evaluated using a modified, validated questionnaire measuring patients’ confidence in performing functional activities aligned with the study’s outcomes.^38 39^ This questionnaire has been validated for the study’s purpose and has been tailored to the study’s functional outcome measures. In addition, a general questionnaire on self-efficacy for arthritis,^40^ including hip OA, will be used. Surgery-related treatment expectations will be assessed using the Stanford Expectations of Treatment Scale (SETS),^41^ while postoperative pain management expectations will be evaluated with the GEEE tool^42^ and NRS scales to assess treatment expectations. Postoperative sickness symptoms and perceived treatment efficacy will be analysed using diverse questionnaires such as the Quality of Recovery-15.^31^ Finally, patients will rate the perceived credibility, warmth and competence of the study physician with the scale developed by Fiske *et al*,^43^ based on the “Stereotype Content Model” (SCM) as well as a specially developed questionnaire and the CARE-D questionnaire.^44 45^

#### Exploratory measures

As part of an exploratory analysis of physiological response measures, blood samples will be collected repeatedly to quantify interleukin-6 (IL-6), C-reactive protein (CRP), erythrocyte sedimentation rate (ESR), and cortisol. These markers are well-established indicators of systemic inflammation and physiological stress and are considered relevant to recovery following orthopaedic surgery.^46^,^48^ Since psychological stress and negative expectations have been linked to elevated inflammatory responses, we aim to explore whether interventions designed to optimise treatment expectations can attenuate biological stress markers. The data will be analysed to explore inflammatory dynamics during both early and post-acute phases, with the aim of identifying potential associations between biological stress responses and subjective reports of pain and sickness symptoms.

The specific parameters assessed are outlined in table 1.

**Table 1:** Outcome measures.

Domain	Measures	Timepoints	Method
Pain	Comparison Pain NRS	T-4, T3, daily during hospital stay	Survey, pain diary
	GCPS^49^	T-4a	Survey
	PDI^50^	T-4a, follow-up	Survey
	GEEE-Effekt^42^	Post T2-T3, follow-up	Survey
	SBI (PHI; Pain Health Inventory)	T-4a / T-4b, post T1-T3, follow-up	Survey
	DN4/DN2^51^	T-4/T1	Examiner / survey
Treatment Expectations	GEEE-Erwartung^42^	T-4a, T-4b	Survey
	SETS^41^	Pre: T-4a, T-4b; post: T2-T3, follow-up	Survey
	Behandlungserfolg PR-OP^8^	T-4a, T-4b, post T1-T2, follow-up	Survey
Mobility	Harris Hip Score^35^	During hospital stay follow-up	Physician examination survey
Patient Physician Relationship	CARE^45^	T-4b, T1	Survey
	Wärme u. Kompetenz^52^	T-4b, T1	Survey
Psychological Trait and State	STADI Trait^53^	T-4a	Survey
	STADI State^53^	T-4a, post T2, follow-up	Survey
	PSS^54^	T-4a; T4	Survey
	SSAS^55^	T-4a; T5	Survey
	FSS^56^	T-4a, post T1	Survey
Postoperative Recovery	QoR-15^36^	Post T1	Survey
	Medication Intake	Daily during hospital stay	Pain diary
Self-efficacy	ASES-D^40^	T-4a/ T-4b, post T1-T3, follow-up	Survey
Systemic inflammation	Laboratory parameter (IL-6, CRP, ESR)	T-4a, post T1, post T3, follow-up	Central clinical laboratory
Physiological stress	Laboratory parameter (cortisol)	T-4a, post T1, post T3, follow-up	Central clinical laboratory

ASES-D, Arthritis Self-Efficacy Scale German version; Behandlungserfolg PR-OP, Perceived Treatment Success Pre-/Post-Op; CARE, Consultation and Relational Empathy Measure; CRP, C-reactive Protein; DN4 / DN2, Douleur Neuropathique 4 / Douleur Neuropathique 2; ESR, Erythrocyte Sedimentation Rate; FSS, Fatigue Severity Scale; GCPS, Graded Chronic Pain Scale; GEEE-Effekt, Göttingen Questionnaire on Experience and Evaluation of Health Care Effect scale; GEEE-Erwartung, Göttingen Questionnaire on Experience and Evaluation of Health Care Expectation scale; Harris Hip Score, Harris Hip Score; IL-6, Interleukin-6; NRS, Numeric Rating Scale; PDI, Pain Disability Index; PSS, Perceived Stress Scale; QoR-15, Quality of Recovery-15; SBI (PHI), Surgical Behavior Inventory (Pain Health Inventory); SETS, Stanford Expectations of Treatment Scale; SSAS, Somatosensory Amplification Scale; STADI Trait / State, State-Trait Anxiety-Depression Inventory; T1, 1 day postoperative; T2, day of discharge; T3, 1-month follow-up; T4, 3-month follow-up; T5, 6-month follow-up; T-4b, 4 days preoperative, after the intervention; Timepoints: T-4a, 4 days preoperative, before the intervention; Wärme u. Kompetenz, Warmth and Competence Scale.

## Sample size calculation

This RCT will include 200 patients undergoing THA due to hip OA. The sample size was determined through an a priori power analysis using G*Power designed to detect interaction effects in a repeated measures ANOVA for the primary outcome of pain intensity. Based on our previous work, we assumed a small to medium effect size of f=0.15 for interaction effects with α=0.05 and 1 - β=0.90, resulting in a required sample size of n=164 (4 groups).^8 10 29^

To account for potential exclusions or dropouts and to enable stratified randomisation by sex/gender into the four study arms while balancing the sex/gender of the patient featured in the video intervention, we aim to recruit a total of n=200 patients, with n=50 per group. Eligibility will be assessed consecutively for all patients scheduled for THA at the UKE (Location: Helios ENDO-Clinic, UKE).

### Statistical analysis

Differences in clinical outcomes between and within groups will be assessed using ANOVA for the various outcome measures. Most outcomes in this study (e.g., pain intensity, functional scores and self-efficacy and inflammatory markers) are continuous and will be analysed as such. A small number of secondary outcomes, however, are categorical (e.g.,analgesic use) and will be analysed using appropriate methods, such as chi-square tests or logistic regression, depending on the data structure. Data analysis will follow the intention-to-treat principle and will be conducted by a researcher blinded to group assignments. If significant main and interaction effects are identified, post hoc analyses (e.g., Least Significant Difference (LSD)) will be performed. The statistical analyses will be carried out using IBM SPSS Statistics software version 27.0 (IBM Corp), with results presented as means and 95% confidence intervals unless stated otherwise. If required, adjustments for deviations from sphericity will be made using Greenhouse-Geisser or Huynh-Feldt corrections for the F-test df. A two-sided p-value of <0.05 will be considered the threshold for statistical significance in all analyses.

Missing data will be handled using multiple imputation, provided that the necessary assumptions are fulfilled. Participants with extensive missing data (e.g., no primary outcome data at any timepoint) will be excluded from the respective analysis. Alongside the intention-to-treat (ITT) approach, we will conduct a per-protocol sensitivity analysis, excluding participants who did not receive the full assigned intervention. This enables us to assess the robustness of results at different levels of complexity.

### Patient and public involvement

Ensuring that patients benefit from the clinical study has been a priority for this project. This study was developed based on the results of a previous study,^10^ and patients were actively involved in the planning phase. They were consulted on the acceptability of the study design, including the use of a condition involving partial disclosure, the comprehensibility and relevance of the questionnaires, and the informational content deemed essential for participants to understand the intervention. Their input was invaluable in shaping the study.

To ensure the validity and efficacy of the video intervention, it was based on the medical history of a patient with primary hip arthroplasty and was performed by two professional actors (male and female video versions). Patient satisfaction with the videos and distributed materials was carefully assessed by conducting interviews with individuals before their surgery in the clinic setting. These patients will not be later enrolled in the study. Regarding physician-patient communication, we refined the provided information based on evidence-based recommendations from previous research in a similar clinical setting and comparable patient cohorts.^8 10^ The interventions consist of two components designed to enhance treatment expectations: (1) an augmented physician-patient conversation, in which the physician provides empathic, competence-signalling communication and explains the patient’s active role in postoperative recovery; and (2) a brief video showing a model patient demonstrating successful coping and functional recovery after hip arthroplasty. Depending on group allocation, participants receive either one of these interventions, both or none (treatment as usual). All materials were developed based on established principles of social learning, self-efficacy theory and expectation modulation in clinical contexts. The tools and materials used in patient interactions were tested beforehand in individuals with lived experience who were not included in the study.

Furthermore, all participating patients will be asked for in-depth feedback on the study materials and design on the completion of their participation. Once the study results are published, they will be shared with all patients who provided written consent.

### Outlook and perspective

This planned study investigates patients' treatment expectations before and after THA, aiming to translate insights from placebo research into clinical practice. Patients undergoing THA often experience a highly vulnerable emotional state, which may significantly influence their treatment expectations. This study tests specific psychological interventions designed to positively modulate these expectations during the perioperative period, thereby targeting this psychological state at a critical moment in the treatment process.

The results will provide insights into how such expectation-focused interventions can be effectively implemented in the context of joint replacement surgery. Furthermore, the findings will provide a foundation for future clinical research exploring the applicability of expectation-based interventions in other chronic pain conditions and analgesic treatment settings. Ultimately, this research may inform the development of more effective, tolerable and cost-efficient strategies for managing postoperative pain, with potential benefits across a broad range of surgical disciplines.

### Ethics and dissemination

The study was approved by the Hamburg Medical Ethics Council (2023–1 01 189-BO-ff). All participants will be obliged to provide written informed consent prior to enrolment. The study findings will be disseminated through peer-reviewed publications, conference presentations and stakeholder engagement. Participants who have provided written consent to receive follow-up information will be sent a summary of the results on completion of the study.

### Trial registration number

German Clinical Trials Register (DRKS00033212).

## Supplementary material

10.1136/bmjopen-2025-108899 online supplemental file 1

## Data Availability

No data are available.
