# Supplementary material for: Optimising preoperative expectations to reduce postoperative pain and disability in total hip arthroplasty: a study protocol for a randomised controlled trial
Source: BMJ Open. 2025 Nov 13;15(11):e108899. doi: 10.1136/bmjopen-2025-108899 (PMC12625907; doi:10.1136/bmjopen-2025-108899)
Supplement: online supplemental file 1 [file bmjopen-15-11-s001.pdf]

Patient:innen - Information zur Studie

Wissenschaftlicher Studientitel:

Präoperative Optimierung von Behandlungserwartungen zur Verringerung von post-operativen Schmerzen und Funktionsbeeinträchtigungen nach einer Hüftoperation

oder allgemein verständlich ausgedrückt:

Verbesserung der Behandlungserwartung vor der Operation, um nach der Hüftoperation weniger Schmerzen und mehr Beweglichkeit zu haben

Sehr geehrte Patientin, sehr geehrter Patient,

im Rahmen der bevorstehenden, geplanten Hüft-Operation möchten wir Sie bitten, an einer Studie teilzunehmen. Unsere Studie ist Teil eines größeren Forschungsverbundes, mit dem Namen Sonderforschungsbereich (SFB) 289 „Treatment Expectation", gefördert durch die Deutsche Forschungsgemeinschaft (DFG). In diesem Rahmen finden auch Kooperationen mit anderen Wissenschaftler:innen statt.

Was wollen wir untersuchen?

Wir wollen in unserem Projekt untersuchen, wie sich die Behandlungserwartungen von Patient:innen, die eine Hüft-Prothese (TEP) erhalten, auf das Schmerzerleben nach der Operation auswirken. Durch die in unserem Studienprotokoll beschriebenen Maßnahmen wollen wir erreichen, dass die Schmerzen nach der Operation geringer sind und die körperliche Funktionskapazität verbessert ist. Dadurch können auch weniger Schmerzmedikamente erforderlich sein.

Welche Annahmen haben wir?

Nach jeder Operation, auch nach einer Hüft-Operation, treten Schmerzen auf, das ist normal. Deswegen erhalten Sie nach einem klinik-internen Schema Schmerzmedikamente.

Schmerzen, die nach der Operation auftreten, können jedoch unterschiedlich stark sein und auch unterschiedlich bewertet werden. Manchmal werden Sie mächtiger erlebt, manchmal schwächer. Sehr selten besteht ein gewisses Risiko, dass nach der Operation Schmerzen anhalten.

Wir gehen davon aus, dass wir positive Behandlungserwartungen, z.B. an eine Schmerzbehandlung, gezielt bei Patient:innen aufbauen können. Wir nehmen an, dass sich eine positive Behandlungserwartung auch positiv auf das Ergebnis der Schmerzbehandlung und den Aufbau der körperlichen Fitness nach der Operation auswirkt. Der Grund hierfür ist, dass Schmerzen zentral verarbeitet werden, d.h. über unser Gehirn beeinflusst werden. Wir gehen davon aus,

dass positive Erwartungen an eine Schmerzbehandlung das körpereigene Opioidsystem (Endorphinsystem; dies ist die körpereigene Möglichkeit der Schmerzlinderung) anregen.

Welches Ziel verfolgen wir in der Studie?

Wir möchten erreichen, dass Sie so wenig wie möglich durch Schmerzen belastet sind und dass sich die Schmerzen nach der Operation schnell wieder zurückentwickeln und Sie so gut es geht wieder Ihre Beweglichkeit aufbauen können. Um dies zu erreichen, möchten wir über die Schmerzmedikamente hinaus weitere Möglichkeiten bereitstellen, damit Sie eine positive Erwartung im Hinblick auf Ihre Behandlung aufbauen und selber etwas tun können, um Ihre Schmerzen zu lindern.

Hierbei handelt es sich um die folgenden 2 Möglichkeiten:

1.) Zum einen möchten wir Ihnen bereits vor der Operation und auch kurz nach der Operation ein ca. 10-minütiges ***Video „positiver Modellpatient"*** zeigen. In dem Video wird ein Patient gezeigt, der bereits seine Hüft-TEP erhalten hat. In dem Video wird vermittelt, welche positiven Auswirkungen seine Behandlungserwartungen auf das Behandlungsergebnis hatten. Das Betrachten eines Videos von einem anderen Patienten, der über seine erfolgreiche Schmerzbehandlung berichtet, zeigte sich bereits in anderen Studien als sehr effektiv. Das positive Video könnte sich auf Ihre Genesung sehr positiv auswirken. In jedem Fall erwarten wir keine negativen Effekte.

2.) Darüber hinaus besteht die Möglichkeit eines zusätzlichen **„zugewandten Arztgespräch"** kurz vor und nach der Operation**.** Der Arzt bzw. die Ärztin wird Sie in Ihrer positiven Behandlungserwartung und Ihrer Selbst-Wirksamkeit, also in dem, was Sie selbst zum Behandlungserfolg beitragen können, durch eine zugewandte informative Arztvisite und kompetentes Auftreten unterstützen und verstärken.

Es gibt bislang noch keinen Nachweis darüber, wie diese beiden Verfahren zusammenwirken. Deshalb möchten wir in vier unterschiedlichen Behandlungsgruppen das ***Video „positiver Modellpatient"*** und das ***„zugewandte Arztgespräch"*** in verschiedenen Kombinationen anwenden.

Zufällige Einteilung („Randomisierung") in die Behandlungsgruppen

Sollten Sie sich entschließen, an dieser vollständig freiwilligen Studie teilzunehmen und die Kriterien für diese Studie erfüllen, dann werden Sie bei einem Telefonat vor der Studie per Zufallsschema zu einer unserer drei Untersuchungsgruppen *oder* der Kontrollgruppe eingeteilt („randomisiert"):

Untersuchungsgruppe 1: Diese Untersuchungsgruppe sieht das ***Video „positiver Modellpatient",*** in dem ein Patient über seine ***positiven*** Erfahrungen mit der Hüft-TEP Operation berichtet erhält ***und ein*** zusätzliches ***„zugewandtes Arztgespräch"***.

Untersuchungsgruppe 2: Diese Untersuchungsgruppe sieht das ***Video „positiver Modellpatient“,*** in dem ein Patient über seine **positiven** Erfahrungen mit der Hüft-TEP Operation berichtet, erhält aber ***kein*** zusätzliches ***„zugewandtes Arztgespräch“.***

Untersuchungsgruppe 3: Diese Untersuchungsgruppe sieht das ***Video „neutraler Modellpatient“.*** Dies ist ein Video, in dem ein Patient über seine Erfahrungen mit der Hüft-TEP Operation berichtet. Zudem erhalten die Patienten und Patientinnen dieser Gruppe ***ein zusätzliches „zugewandtes Arztgespräch“.***

Untersuchungsgruppe 4 (Kontrollgruppe): Diese Untersuchungsgruppe sieht das ***Video „neutraler Modellpatient“,*** in dem ein Patient über seine Erfahrungen mit der Hüft-TEP Operation berichtet ***und*** erhält ***kein*** zusätzliches ***„zugewandtes Arztgespräch“.***

Unabhängig davon, welcher Behandlungsgruppe Sie zugeteilt werden, oder unabhängig davon, ob Sie sich entscheiden an dieser Studie teilzunehmen oder nicht, werden Sie während des Aufenthaltes durch unser Schmerzteam bei Schmerzen rund um die Uhr betreut und bei auftretenden Schmerzen adäquat mit der Gabe von Schmerzmitteln versorgt. Durch die Teilnahme an der Studie entstehen Ihnen keine Nachteile in Ihrer Behandlung und Ihrer Schmerzbehandlung.

Sie werden im Rahmen dieser Studie keine zusätzlichen Schmerzen erleiden. Abgesehen von einer zusätzlichen Betreuung vor und nach der Operation hat diese Studie keinerlei Einfluss auf Ihre Behandlung, Narkoseführung, Operation oder Entscheidungsfindung. Neue Medikamente oder Verfahren werden nicht getestet.

Ablauf der Studie und Untersuchungsaufwand

Wenn Sie sich entschließen, an unserer Studie teilzunehmen, dann kommt ein zeitlicher Aufwand für Befragung, Video betrachten, Untersuchungen von ca. 7 Stunden auf Sie zu, und zwar ***vor*** (eine Woche vor der Operation), ***während*** (bei Aufnahme, am 1. Tag nach der Operation) und auch noch ***2 x nach*** Ihrem stationären Aufenthalt (3 und 6 Monate später).

1.) *Fragebögen:* Zum einen werden wir Sie bitten, uns Fragebögen über Ihre Erwartungen, Stimmung, Zufriedenheit, Schmerzen und Beweglichkeit auszufüllen, eine Woche vor der Operation, während der stationären Aufnahme und 3 und 6 Monate später. Zudem erhalten Sie während des stationären Aufenthaltes ein vorgefertigtes Schmerztagebuch. Dies sollte täglich nicht mehr als 15 Minuten Ihrer Zeit beanspruchen. Alle Daten der Fragebögen werden pseudonymisiert, d.h. dass die Ergebnisse der Befragung nur durch autorisierte Personen Ihrer Person zugeordnet werden können.
2.) *Blutuntersuchungen:* zur Bestimmung bestimmter Veränderung der postoperativen Entzündungsparameter werden Ihnen im Rahmen der Studie zusätzlich jeweils 10 Milliliter Blut vor (Narkosevorgespräch und Operation) und nach dem operativen Eingriff auf Station täglich und nochmals 3 und 6 Monate später abgenommen.

Die Ergebnisse der Blutuntersuchung werden bis zur Analyse pseudonymisiert gespeichert und ausgewertet und nach den Analysen sicher vernichtet.

Gibt es für mich gesundheitliche Risiken, die mit der Teilnahme an der Studie verbunden sind?

Im Zentrum unserer Studie steht die Untersuchung Ihrer Erwartungen gegenüber Ihrer Schmerzbehandlung, die Sie in unserer Klinik erhalten. Mit den Behandlungen sind keine gesundheitlichen Risiken verbunden.

Es wird vor der Behandlung genau ärztlicherseits geprüft, ob diese für Sie geeignet sind, d.h. ob die Indikation dafür besteht oder nicht. Es werden zusätzlich Blutproben entnommen. Das Risiko der durchgeführten Blutentnahmen entspricht dem, was Sie von Blutentnahmen bei einem regulären Arztbesuch kennen. Bei Blutentnahmen kann es in seltenen Fällen zu Nervenverletzungen kommen.

Die Studie ist freiwillig, d.h. Sie haben zu jeder Zeit die Möglichkeit, die Studie abzubrechen. Sollte eine unerwartete Schmerzverstärkung auftreten, dann werden Sie sofort weiter versorgt. Auch eine Änderung der Medikation ist zu jedem Zeitpunkt möglich.

Ergänzende Information für Studienteilnehmer gemäß Europäischer Datenschutz-Grundverordnung[1]

Wissenschaftlicher Studientitel:

Präoperative Optimierung von Behandlungserwartungen zur Verringerung von postoperativen Schmerzen und Behinderungen nach einer Hüftoperation

oder allgemein verständlich ausgedrückt:

Verbesserung der Behandlungserwartung vor der Operation, um nach der Hüftoperation weniger Schmerzen und mehr Beweglichkeit zu haben

Sehr geehrte Patientin, sehr geehrter Patient,

aufgrund des Wirksamwerdens der Europäischen Datenschutz-Grundverordnung (**DSGVO**) zum 25. Mai 2018, ändern sich die Datenschutzvorschriften in Europa. Für klinische Studien ergeben sich dadurch neue Anforderungen an die Verarbeitung personenbezogener Daten. Dies beinhaltet z. B. Informationen über die Erfassung, Speicherung und Weiterleitung Ihrer personenbezogenen Daten sowie Ihre diesbezüglichen Rechte. Auch als mögliche/r neue/r Studienteilnehmer/in erhalten Sie diese Informationen im Rahmen des Aufklärungsgesprächs durch Ihren Prüfer und in der schriftlichen Patienteninformation- und Einwilligungserklärung zur klinischen Studie. Der in der Patienteninformation- und Einwilligungserklärung zu der jeweiligen klinischen Studie beschriebene Umgang mit Ihren Daten gilt weiterhin.

Pseudonymisierte und Anonymisierte Daten

Bei den pseudonymisierten Daten werden Identifikationsmerkmale (z.B. Name) durch einen Code ersetzt, der nur durch einen bestimmten Schlüssel wiederhergestellt werden kann. Dabei bleibt eine Verbindung zwischen den Daten und Ihnen bestehen, jedoch hat ausschließlich die Studienleitung Zugriff der Codierungsliste und Ihren persönlichen Daten, die im Studienzentrum hinterlegt ist. Diese Liste wird im Studienzentrum gesondert aufbewahrt und unterliegt dort technischen und organisatorischen Maßnahmen, die gewährleisten, dass die personenbezogenen Daten Ihnen durch unbefugte Personen nicht zugeordnet werden können. Eine Entschlüsselung erfolgt nur für den Zweck, dass weitere Daten für das Projekt

---

[1] Verordnung (EU) 2016/679 des Europäischen Parlaments und des Rates vom 27. April 2016 zum Schutz natürlicher Personen bei der Verarbeitung personenbezogener Daten, zum freien Datenverkehr und zur Aufhebung der Richtlinie 95/46/EG (Datenschutz-Grundverordnung)

erhoben werden müssen (z.B. der Zugriff auf Ihren Namen und Telefonnummer, um einen Nachkontrolluntersuchungstermin im Rahmen des Forschungsprojektes zu vereinbaren).
Bei den anonymisierten Daten werden alle Identifikationsmerkmale entfernt, sodass keine Verbindung zwischen Ihnen und den Daten hergestellt werden kann. Uns ist es außerordentlich wichtig, dass Ihre Vertraulichkeit und Privatsphäre gewahrt wird.

Risiken Datenverarbeitung

Bei jeder Erhebung, Speicherung, Nutzung und Übermittlung von Daten bestehen Vertraulichkeitsrisiken (z.B. die Möglichkeit, die betreffende Person zu identifizieren). Diese Risiken lassen sich nicht völlig ausschließen und steigen, je mehr Daten miteinander verknüpft werden können. Der Initiator der Studie versichert Ihnen, alles nach dem Stand der Technik Mögliche zum Schutz Ihrer Privatsphäre zu tun und Daten nur an Stellen weiterzugeben, die ein geeignetes Datenschutzkonzept vorweisen können. Medizinische Risiken sind mit der Datenverarbeitung nicht verbunden.
Die anonymisierten Daten können auch in Länder außerhalb des EU-Binnenraumes weitergegeben werden, z.B. Niederlande, Dänemark. In diesen Ländern besteht möglicherweise ein geringeres Datenschutzniveau. Mit Ihrer Einwilligung stimmen Sie zu, dass die anonymisierten Daten auch in diese Länder übermittelt werden dürfen.

Zusätzlich werden Sie hiermit über die in der DSGVO festgelegten Rechte informiert (Artikel 12 ff. DSGVO)**:**

Rechtsgrundlage

Die Rechtsgrundlage zur Verarbeitung der Sie betreffenden personenbezogenen Daten bildet bei klinischen Studien Ihre freiwillige schriftliche Einwilligung gemäß Art. 6 Abs. 1 Satz 1 lit. a, Art. 9 Abs. 2 lit. a DSGVO; sowie der Deklaration von Helsinki (Erklärung des Weltärztebundes zu den ethischen Grundsätzen für die medizinische Forschung am Menschen) und der Leitlinie für Gute Klinische Praxis. Zeitgleich mit der DSGVO tritt in Deutschland das überarbeitete Bundesdatenschutzgesetz (BDSG-neu) in Kraft.

Für die Datenverarbeitung verantwortliche Person

Die Studienleiterin des Universitätsklinikums Hamburg-Eppendorf ist Frau PD Dr. Regine Klinger.

Recht auf Auskunft

Sie haben das Recht auf Auskunft über die Sie betreffenden personenbezogenen Daten, die im Rahmen der klinischen Studie erhoben, verarbeitet oder ggf. an Dritte übermittelt werden (Aushändigen einer kostenfreien Kopie) (Artikel 15 DSGVO, §34 BDSG-neu).

Recht auf Berichtigung

Sie haben das Recht, Sie betreffende unrichtige personenbezogene Daten berichtigen zu lassen (Artikel 16 und 19 DSGVO).

Recht auf Löschung
Sie haben das Recht auf Löschung Sie betreffender personenbezogener Daten, z. B. wenn diese Daten für den Zweck, für den sie erhoben wurden, nicht mehr notwendig sind (Artikel 17 und 19 DSGVO, §35 BDSG-neu).

Recht auf Einschränkung der Verarbeitung
Unter bestimmten Voraussetzungen haben Sie das Recht, eine Einschränkung der Verarbeitung zu verlangen, d.h. die Daten dürfen nur gespeichert, aber nicht verarbeitet werden. Dies müssen Sie beantragen. Wenden Sie sich hierzu bitte an Ihren Studienleiter oder an den Datenschutzbeauftragten des Prüfzentrums (Artikel 18 und 19 DSGVO).

Recht auf Datenübertragbarkeit
Sie haben das Recht, die Sie betreffenden personenbezogenen Daten, die Sie dem Verantwortlichen für die klinische Studie bereitgestellt haben, zu erhalten. Damit können Sie beantragen, dass diese Daten entweder Ihnen oder, soweit technisch möglich, einer anderen von Ihnen benannten Stelle übermittelt werden (Artikel 20 DSGVO).

Widerspruchsrecht
Sie haben das Recht, jederzeit gegen konkrete Entscheidungen oder Maßnahmen zur Verarbeitung der Sie betreffenden personenbezogenen Daten Widerspruch einzulegen (Art 21 DSGVO, § 36BDSG-neu). Eine solche Verarbeitung findet anschließend grundsätzlich nicht mehr statt.

Einwilligung zur Verarbeitung personenbezogener Daten und Recht auf Widerruf dieser Einwilligung
Die Verarbeitung Ihrer personenbezogenen Daten ist nur mit Ihrer Einwilligung rechtmäßig (Artikel 6 DSGVO). Sie haben das Recht, Ihre Einwilligung zur Verarbeitung personenbezogener Daten jederzeit zu widerrufen. Im Falle des Widerrufs müssen Ihre personenbezogenen Daten grundsätzlich gelöscht werden (Artikel 7, Absatz 3 DSGVO). Es gibt allerdings Ausnahmen, nach denen die bis zum Zeitpunkt des Widerrufs erhobenen Daten weiterverarbeitet werden dürfen, z.B. wenn die weitere Datenverarbeitung zur Erfüllung einer rechtlichen Verpflichtung erforderlich ist (Art. 17 Abs. 3 b DSGVO).

Möchten Sie eines dieser Rechte in Anspruch nehmen, wenden Sie sich bitte an die Studienleiterin Ihres Prüfzentrums. Außerdem haben Sie das **Recht, Beschwerde bei den Aufsichtsbehörden einzulegen,** wenn Sie der Ansicht sind, dass die Verarbeitung der Sie betreffenden personenbezogenen Daten gegen die DS-GVO verstößt:

Studienleitung

PD Dr. Regine Klinger
Psychologische Leiterin
des Bereichs Schmerzmedizin und Schmerzpsychologie
Universitätsklinikum Hamburg-Eppendorf (UKE)
Zentrum für Anästhesiologie und Intensivmedizin
Klinik und Poliklinik für Anästhesiologie
Neues Klinikum O10, Raum 02.5.045.1
Martinistraße 52
20246 Hamburg
040 / 7410 - 20130
r.klinger@uke.de

Datenschutz: Kontaktdaten der Aufsicht der Studienleitung

**Datenschutzbeauftragte/r**	**Datenschutz-Aufsichtsbehörde**
Datenschutzbeauftragter des Universitätsklinikums Hamburg-Eppendorf	Der Landesbeauftragte für den Datenschutz Hamburg
Martinistraße 52	Klosterwall 6
20246 Hamburg	20095 Hamburg
040 / 7410 - 56890	040 / 42854 - 4040
m.jaster@uke.de	mailbox@datenschutz.hamburg.de

Studienleiterin: PD Dr. Regine Klinger; Tel.: +49 40 7410 - 20130

Einwilligungserklärung zur Teilnahme an der Studie

Wissenschaftlicher Studientitel:

Präoperative Optimierung von Behandlungserwartungen zur Verringerung von postoperativen Schmerzen und Behinderungen nach einer Hüftoperation

oder allgemein verständlich ausgedrückt:

Verbesserung der Behandlungserwartung vor der Operation, um nach der Hüftoperation weniger Schmerzen und mehr Beweglichkeit zu haben

Name des/der Patient:in in Druckbuchstaben: ……………………………

- Ich bin von Herrn / Frau ________________ über Wesen, Bedeutung und Tragweite der Studie sowie die sich für mich daraus ergebenden Anforderungen aufgeklärt worden. Ich habe darüber hinaus den Text der Patientenaufklärung und dieser Einwilligungserklärung gelesen.
- Ich hatte ausreichend Zeit, Fragen zu stellen und mich zu entscheiden. Aufgetretene Fragen wurden mir vom Studienarzt beantwortet.
- Ich weiß, dass ich meine freiwillige Mitwirkung jederzeit beenden kann, ohne dass mir daraus Nachteile entstehen.

Ich erkläre mich bereit, an der Studie teilzunehmen.

1. Ich willige ein, dass personenbezogene Daten über mich, insbesondere Vor-/Nachname, Adresse, Geburtsdatum, Diagnosen, Fragebögen, Physiologische Daten, wie in der Informationsschrift beschrieben, erhoben und in Papierform sowie auf elektronischen Datenträgern im UKE, Hamburg, sowie am Standort Helios ENDO-Klinik, aufgezeichnet und ausgewertet werden. Zu diesem Zweck entbinde ich die mich behandelnden Ärzte/Psychologen von der ärztlichen/psychologischen Schweigepflicht.

Soweit erforderlich, dürfen die erhobenen Daten pseudonymisiert (verschlüsselt) weitergegeben werden:

a) an Mitglieder der Forschungsgruppe SFB 289 zum Zweck der wissenschaftlichen Auswertung,

b) im Falle unerwünschter Ereignisse: an die jeweils zuständige Ethik-Kommission und zuständige Behörden sowie von dieser an die Europäische Datenbank.

2. Ich willige ein, dass mir Blutproben zur Bestimmung von Zellzahlen, Entzündungsmarkern sowie zur wissenschaftlichen Untersuchung mögliche Biomarker von akuten Schmerzen entnommen werden. Die hierbei erhobenen Daten dürfen gemäß Punkt 1. verarbeitet und weitergegeben werden.

3. Ich bin darüber aufgeklärt worden, dass ich meine Einwilligung jederzeit widerrufen kann. Im Falle des Widerrufs werden keine weiteren Daten mehr erhoben. Ich kann in diesem Fall die Löschung der Daten verlangen, sofern diese noch nicht anonymisiert worden sind. Anonymisierte Daten können nicht gelöscht werden, da sie nicht mehr einer Person zuzuordnen sind.

4. Ich willige ein, dass der „Code-Schlüssel“, der eine Zuordnung des Codes zu Ihren personenbezogenen Daten ermöglicht für die Laufzeit der Datenerhebung und bis zu dem Zeitpunkt, an dem die Daten der Studie ausgewertet sind, aufgehoben wird.

5. Ich bin darüber aufgeklärt worden, dass die anonymisierten Daten nach Beendigung und Veröffentlichung von Studienergebnissen mindestens 10 Jahre aufbewahrt werden.

Kontaktdaten der Studienleitung:

PD Dr. Regine Klinger
Universitätsklinikum Hamburg-Eppendorf (UKE)
Zentrum für Anästhesiologie und Intensivmedizin
Klinik und Poliklinik für Anästhesiologie
Neues Klinikum O10
Martinistraße 52
20246 Hamburg
r.klinger@uke.de
Tel: 040 7410 20130

Ich willige in die Verarbeitung der genannten Daten ein.

Ein Exemplar der Informationsschrift und der Einwilligungserklärung habe ich erhalten. Ein Exemplar verbleibt im Prüfzentrum.

Unterschrift des Teilnehmers / der Teilnehmerin

____

(Name und Vorname in Druckschrift)

___________________________________ ___________________________

(Datum) (Unterschrift)

Erklärung und Unterschrift der/des aufklärenden Arzt/Ärztin oder Psychologe/Psychologin

Ich habe das Aufklärungsgespräch geführt und die Einwilligung eingeholt.

____

(Name und Vorname in Druckschrift)

___________________________________ ___________________________

(Datum) (Unterschrift)

Patients – Information about the study

Scientific titel of the study:

Preoperative optimization of treatment expectations to reduce postoperative pain and functional impairment after hip surgery

or in general terms:

Improving treatment expectations before surgery in order to experience less pain and greater mobility after hip surgery

Dear patient,

As part of your upcoming hip surgery, we would like to invite you to take part in a research study. This study is part of a larger research network called the Collaborative Research Centre (CRC) 289, which is funded by the German Research Foundation (DFG) and focuses on 'Treatment Expectation'. Collaborations with other researchers also take place in this context.

What do we want to investigate?

The aim of our project is to investigate how the treatment expectations of patients undergoing total hip arthroplasty (THA) influence their experience of pain after surgery. Through the measures described in our study protocol, we intend to minimise postoperative pain and enhance physical functioning. Consequently, the need for pain medication may also decrease.

What assumptions do we have?

It is normal to experience pain after any surgery, including hip surgery. For this reason, you will be given pain medication in accordance with the hospital's standard protocol.

However, the intensity and perception of postoperative pain can vary. Sometimes it is perceived as stronger and at other times as milder. In rare cases, there is a risk that the pain may persist after the operation.

We believe that positive treatment expectations can be deliberately fostered in patients, for example with regard to pain management. We believe that positive expectations can have a positive influence on the outcome of pain therapy and the recovery of physical functioning after surgery. The underlying reason is that pain is processed centrally, meaning it is influenced by the brain. We hypothesise that positive expectations regarding pain treatment can activate the body's endogenous opioid system, which is responsible for natural pain relief.

What is the aim of the study?

Our goal is to ensure that you experience minimal pain, that any postoperative pain subsides quickly and that you regain your mobility effectively. To achieve this, we would like to offer you additional options in order to help you develop a positive expectation regarding your treatment and to empower you to actively contribute to your own pain relief.

There are two options availble:

1.) First, we like to show you a short video, which is approximately 10 minutes long, of what happens both before and shortly after the operation. The video features a **„positive role model patient“** who has already undergone total hip arthroplasty. It illustrates how positive treatment expectations can contribute to a successful recovery. Previous studies have shown that watching a video in which another patient reports on their successful pain treatment is highly effective. We believe that this positive video could have a beneficial impact on your recovery. In any case, we do not anticipate any negative effects.

2.) In addition, you may receive a **„supportive physician consultation“** shortly before and after the operation. During this consultation, the doctor will encourage positive expectations of treatment and self-efficacy — that is, confidence in your ability to contribute to the success of your treatment — by providing clear, supportive medical information and demonstrating confidence and compassion.

Currently, there is no evidence on how these two approaches work in combination. For this reason, we plan to use the **„positive role model patient“** video and the 'supportive physician consultation' with four different treatment groups, in various combinations.

Random allocation to treatment groups

If you decide to participate in this entirely voluntary study and meet the eligibility criteria, you will be randomly assigned to one of three intervention groups or the control group during a telephone call prior to the study (“randomisation”):

Intervention group 1: Participants in this group will watch the **„positive role model patient“ video**, in which a patient shares their positive experience of hip replacement surgery. They will also receive an additional **„supportive consultation“** with a physician.

Intervention group 2: Participants in this group will watch the **„positive role model patient“** video, in which a patient shares their positive experience of hip replacement surgery. However, they will **not** receive the additional **„supportive consultation“** with a physician.

Intervention group 3: Participants in this group will not watch the **„positive role model patient“** video, in which a patient shares their experiences with hip replacement surgery. They will receive a **„supportive physician consultation“.**

Intervention group 4 (Control group): Participants in this group will **not** receive a **video** or a **„supportive physician consultation“.** These patients receive treatment as usual.

Regardless of which treatment group you are assigned to, or whether you choose to participate in the study, you will receive continuous support from our pain management team throughout your hospital stay. You will receive appropriate pain medication as required.Taking part in this study will not affect your medical care or pain management in any way.

Your participation in this study will not result in any additional pain. Apart from the extra care you will receive before and after the surgery, the study will not affect your treatment, anaesthesia, surgery or the decision-making process. No new medications or procedures will be tested.

Study procedure and examination effort

If you decide to participate in our study, you should expect to spend approximately 7 hours completing questionnaires, watching videos and taking assessments. These will take place before surgery (approximately four days beforehand), during your hospital stay (the day after surgery and discharge day) and twice after discharge (one, three and six months afterwards).

1.) *Questionnaires:* We will ask you to complete questionnaires about your expectations, mood, satisfaction, pain, and mobility—four days before the operation, during your hospital stay, and again at 1, 3 and 6 months after the surgery. In addition, you will receive a pre-structured pain diary during your hospital stay. Completing the diary should take no more than 15 minutes per day. All questionnaire data will be pseudonymised, meaning that the results can only be linked to your identity by authorised personnel.
2.) *Laboratory markers:* To assess changes in postoperative inflammatory markers, we will take an additional 10 milliliters of blood from you as part of the study—before the operation (during the pre-anaesthesia consultation), during your hospital stay (two days after the surgery), and again at 3 and 6 months after the procedure.

The results of the blood test are stored and evaluated in pseudonymised form until they have been analysed, and are then securely destroyed after analysis.

Studienleiterin: PD Dr. Regine Klinger; Tel.: +49 40 7410 - 20130

Are there any health risks for me associated with participating in the study?

The focus of our study is investigating your expectations of the pain treatment you will receive at our clinic. These treatments carry no health risks.

Before any treatment is administered, a physician will carefully assess whether it is appropriate for you, i.e. whether the indication is given. Additional blood samples will be taken. The risks associated with these blood tests are similar to those of a routine blood test during a regular doctor's appointment. In rare cases, blood sampling may cause nerve injury.

Participation in the study is voluntary, which means that you can withdraw at any time without facing any consequences. If you experience any unexpected increases in pain, you will receive immediate medical care. Any necessary medication adjustments can be made at any time.

Studienleiterin: PD Dr. Regine Klinger; Tel.: +49 40 7410 - 20130

Supplementary information for study participants in accordance with the European General Data Protection Regulation[1]

Scientific titel of the study:

Preoperative optimization of treatment expectations to reduce postoperative pain and functional impairment after hip surgery

or in general terms:

Improving treatment expectations before surgery in order to experience less pain and greater mobility after hip surgery

Dear patient,

The entry into force of the European General Data Protection Regulation (GDPR) on 25 May 2018 has changed data protection regulations in Europe. This has resulted in new requirements for the processing of personal data in clinical studies. These include information about the collection, storage and transfer of your personal data, and your associated rights. As a potential new study participant, you will receive this information during your consent discussion with the study physician, as well as in the written patient information and consent form for the clinical study. The procedures for handling your data, as described in the patient information and consent form for the relevant study, will continue to apply.

Pseudonymised and anonymised data

Pseudonymised data replaces identifying information (e.g. name) with a code that can only be traced back using a specific key. Although a link between the data and your identity remains, only the study management team has access to the coding list and your personal information, which is securely stored at the study site. This list is stored separately at the study centre and protected by technical and organisational measures to prevent unauthorised persons from linking the data to your identity. Decoding will only occur if additional data needs to be collected for the project (for example, if we need to access your name and telephone number to schedule a follow-up visit as part of the research project).
For anonymised data, all identifying information is removed so that no connection can be made between you and the data.
We place the utmost importance on protecting your confidentiality and privacy.

Risks of data processing

Every instance of data collection, storage, use and transmission carries some risk to confidentiality, for example the possibility of identifying the individual concerned. These risks cannot be completely eliminated, especially when multiple data sets are linked.

[1] Verordnung (EU) 2016/679 des Europäischen Parlaments und des Rates vom 27. April 2016 zum Schutz natürlicher Personen bei der Verarbeitung personenbezogener Daten, zum freien Datenverkehr und zur Aufhebung der Richtlinie 95/46/EG (Datenschutz-Grundverordnung)

However, the study sponsor assures you that all technically feasible measures will be taken to protect your privacy, and that data will only be shared with parties that can demonstrate an appropriate data protection framework. There are no medical risks associated with data processing. Anonymised data may also be transferred to countries outside the European Union, such as the Netherlands and Denmark. These countries may have a lower level of data protection. By providing your consent, you agree to the transfer of anonymised data to these countries.

In addition, you are hereby informed of your rights as set out in the GDPR (Article 12 et seq. GDPR):

Legal framework

The legal basis for the processing of your personal data in clinical studies is your voluntary written consent in accordance with Article 6(1)(a) and Article 9(2)(a) of the General Data Protection Regulation (GDPR); as well as the Declaration of Helsinki (World Medical Association's statement of ethical principles for medical research involving human subjects) and the International Council for Harmonisation Guideline for Good Clinical Practice (ICH-GCP). At the same time as the GDPR, the revised Federal Data Protection Act (BDSG-new) also comes into force in Germany.

Data controller

The study director at the University Medical Centre Hamburg-Eppendorf is Dr Regine Klinger.

Right to access

You have the right to access the personal data concerning you that is collected, processed, or potentially transferred to third parties in the course of the clinical study (including the provision of a free copy) (Article 15 GDPR, §34 BDSG-new).

Right to rectification

You have the right to request the rectification of inaccurate personal data concerning you (Articles 16 and 19 GDPR).

Right to erasure

You have the right to request the deletion of your personal data, for example if the data are no longer necessary for the purposes for which they were collected (Articles 17 and 19 GDPR, §35 BDSG-new).

Right to restriction of processing

Under certain conditions, you have the right to request a restriction of processing, meaning that your data may only be stored but not further processed. You must submit a request for this. Please contact your study physician or the data protection officer at the study center (Articles 18 and 19 GDPR).

Right to data portability

You have the right to receive the personal data concerning you that you have provided to the controller of the clinical study. You may request that these data be transmitted either to you or, where technically feasible, to another party designated by you (Article 20 GDPR).

Right of objection

You have the right to object at any time to specific decisions or measures concerning the processing of your personal data (Article 21 GDPR, §36 BDSG-new). Following such an objection, this processing will generally no longer take place.

Consent to the processing of personal data and right to withdraw this consent

The processing of your personal data is lawful only with your consent (Article 6 GDPR). You have the right to withdraw your consent to the processing of personal data at any time. In the event of withdrawal, your personal data must generally be deleted (Article 7(3) GDPR). However, there are exceptions allowing continued processing of data collected up to the time of withdrawal—for example, when further processing is required to comply with a legal obligation (Article 17(3)(b) GDPR).

If you wish to exercise any of these rights, please contact the study director at your trial centre. You also have the right to lodge a complaint with the supervisory authorities if you believe that the processing of your personal data violates the GDPR:

Study management

PD Dr. Regine Klinger
Psychological Director
of the field of pain medicine and pain psychology
University Medical Center Hamburg-Eppendorf (UKE)
Centre for Anaesthesiology and Intensive Care Medicine
Clinic and Polyclinic for Anaesthesiology
Main building O10, Raum 02.5.045.1
Martinistraße 52
20246 Hamburg
040 / 7410 - 20130
r.klinger@uke.de

Data protection: Contact details of the study management supervisory authority

**Data protection officer**	**Data protection supervisory authority**
Data protection officer	The State Commissioner for data protection
University Medical Center	in Hamburg

Hamburg-Eppendorf
Martinistraße 52
20246 Hamburg
040 / 7410 - 56890
m.jaster@uke.de

Klosterwall 6
20095 Hamburg
040 / 42854 - 4040
mailbox@datenschutz.hamburg.de

Studienleiterin: PD Dr. Regine Klinger; Tel.: +49 40 7410 - 20130

Declaration of consent to participate in the study

Scientific titel of the study:

Preoperative optimization of treatment expectations to reduce postoperative pain and functional impairment after hip surgery

or in general terms:

Improving treatment expectations before surgery in order to experience less pain and greater mobility after hip surgery

Patient´s name in block letters: ....

- I have been informed by Mr/Ms _______________ about the nature, significance and scope oft he study and the requirements that entails for me. I have also read the patient information sheet and this consent form.
- I had sufficient time to ask questions and make a desicion. Any questions I had were answered by the study doctor.
- I know that I can terminate my voluntary participation at any time without incurring any disadvantages.

I agree to participate to the study.

1. I consent to the collection, recording and evaluation of personal data about me, in particular my first name, surname, address, date of birth, diagnoses, questionnaires and physiological data, as described in the information leaflet, in paper form and on electronic data carriers at the UKE, Hamburg, and at the Helios ENDO Clinic. For this purpose, I release the doctors/psychologists treating me from their medical/psychological duty of confidentiality.

Where necessary, the data collected my be passed on in pseudonymised (encrypted) form:

a) tot he members of the SFB 289 research group fort he purpose of scientific evaluation,

b) in the case of adverse events: tot he relevant ethics committee and competent authorities, and from there tot he European database.

2. I consent to blood samples being taken from me to determine cell counts and inflammation markers and for scientific investigation of possible biomarkers of acute pain. The data collected in this process may be processed and passed on in accordance with point 1.

3. I have been informed that i can revoke my consent at any time. In the event of revocation, no further data will be collected. In this case, I can request the deletion of the data, provided that it has not yet been anonymised. Anonymised data cannot be deleted, as it can no longer be attributed to a specific person.

4. I consent to the „code key“ that enables the code to be assigned to your personal data being retained for the duration of the data collection and until the data from the study has been evaluated.

5. I I have been informed that the anonymised data will be stored for at least 10 years after the completion and publication of the study results.

Contact details for the study director:

PD Dr. Regine Klinger
University Medical Center Hamburg-Eppendorf
Centre for Anaesthesiology and Intensive Care Medicine
Clinic and Polyclinic for Anaesthesiology
Main building O10
Martinistraße 52
20246 Hamburg
r.klinger@uke.de
Tel: 040 7410 20130

I consent to the processing of the aforementioned data.

I have received a copy of the information sheet and the consent form. One copy remains at the test centre.

Signature of the participant

____

(Surname and first name in block letters)

___________________________________ ___________________________

(Date) (Signature)

Declaration and signature of the doctor or psychologist who provided the information

I conducted the consultation and obtained consent.

____

(Surname and first name in block letters)

___________________________________ ___________________________

(Date) (Signature)
