# Supplementary material for: Optimising preoperative expectations to reduce postoperative pain and disability in total hip arthroplasty: a study protocol for a randomised controlled trial
Source: BMJ Open. 2025 Nov 13;15(11):e108899. doi: 10.1136/bmjopen-2025-108899 (PMC12625907; doi:10.1136/bmjopen-2025-108899)
Supplement: Supplementary file 2 [file bmjopen-2025-108899.reviewer_comments.pdf]

PEER REVIEW HISTORY

BMJ Open publishes all reviews undertaken for accepted manuscripts. Reviewers are asked to complete a checklist review form and are provided with free text boxes to elaborate on their assessment. These free text comments are reproduced below.

ARTICLE DETAILS

Title (Provisional)

Optimising preoperative expectations to reduce postoperative pain and disability in total hip arthroplasty: A study protocol for a randomized controlled trial.

Authors

Ocokoljic , Ana; Stuhlreyer, Julia; Paschke-Dahl, Lena; Elsenbruch, Sigrid; Rolvien, Tim; Beil, Frank Timo; Zöllner, Christian; Klinger, Regine

---

VERSION 1 - REVIEW

Reviewer 1

Name Naylor, Justine

Affiliation Ingham Institute for Applied Medical Research, Whitlam Orthopaedic Research Centre

Date 03-Sep-2025

COI I have just completed a QI study looking at the effect of exposing people to a dose of reality pre arthroplasty on expectations. We have also designed a related longitudinal study.

The manuscript describes a study protocol re: a RCT to improve patient expectations pre-op in order to improve outcomes post-op.

The design is robust and the study fills a research gap.

TITLE

There is an assumption that the intervention will optimize expectations. In Methods, ideally you would state which direction expectations scores will go in to reflect optimization.

ABSTRACT

1. LINE 15 - It is not clear why these things are crucial. Rising numbers does not in itself make these things crucial. Re-phrase

2. Consider changing abbreviation for DOC group or change it to mean “augmented doctor conversation” to make abbreviation make sense?

BACKGROUND

• LINE 17, page 5 – What considerable risk? Is it not mentioned prior to this.

• Suggest line 58, page 5, is not a new paragraph

• Page 6 line 19 - be consistent with terms hip replacement or hip arthroplasty? (and page 16, line 51)

• The review of what is known/has been done re: THA is a little light. Could be bolstered. Much of what is cited relates to theory of expectations/learnings et from elsewhere with little reference to what is known specifically in THA. A quick search of literature identified these papers that I don’t think have been cited. It is clear from below list, not much has been done in this space, thus the study is warranted.

• Mooiweer Y, Roling L, Vugrin M, Ansmann L, Stevens M, Seeber GH. Influence of patients' preoperative expectations on postoperative outcomes after total knee or hip arthroplasty: a systematic review. EFORT Open Rev. 2024 Feb 1;9(2):107-118. doi: 10.1530/EOR-23-0087.

• Tilbury C, Haanstra TM, Verdegaal SHM, Nelissen RGHH, de Vet HCW, Vliet Vlieland TPM, Ostelo RW. Patients' pre-operative general and specific outcome expectations predict postoperative pain and function after total knee and total hip arthroplasties. Scand J Pain. 2018 Jul 26;18(3):457-466. doi: 10.1515/sjpain-2018-0022.

• Neuprez A, Delcour JP, Fatemi F, Gillet P, Crielaard JM, Bruyère O, Reginster JY. Patients' Expectations Impact Their Satisfaction following Total Hip or Knee Arthroplasty. PLoS One. 2016 Dec 15;11(12):e0167911. doi: 10.1371/journal.pone.0167911.

• Laferton JAC, Oeltjen L, Neubauer K, Ebert DD, Munder T. The effects of patients' expectations on surgery outcome in total hip and knee arthroplasty: a prognostic factor meta-analysis. Health Psychol Rev. 2022 Mar;16(1):50-66. doi: 10.1080/17437199.2020.1854051. Epub 2020 Dec 21. PMID: 33228474.

• Mancuso CA, Sculco TP, Salvati EA. Patients with poor preoperative functional status have high expectations of total hip arthroplasty. J Arthroplasty 2003;18 (7):872-878.

METHODS

1. Page 8, line 19-24. It is unclear what is meant by “projects” can a different term be used or explain? Central projects vs participating projects?

2. Page 9 exclusion criteria - clarify what is meant by secondary THA (revision or had previous THA on other side?) Note- prior primary THA (on contralateral side) could be a modifier. In a related thought, assume people cant be enrolled in the study twice Eg if come back for 2nd joint within study time frame

3. Page 9, exclusion criteria - How is opioid use determined and classified eg any use or daily use or dose dependent?

4. Page 9, exclusions – why is pregnancy/breast feeding an issue? Normally explain when it is unusual (would the interventions affect these or doe the assessments contraindicate these) (I note people who are pregnant/breast-feeding are not normally undergoing major arthroplasty anyway so not usually stated)

5. Page 10 line 3 - when pre-surgery is this visit? Clarify here as this is when randomization takes place and we need to know time from b/w randomization and intervention delivery (I note this is clarified in 2.4 but needs to be stated here instead)

6. Page 10, line 45 - are German THA patents really on average 60 yrs old? This seems relatively young.

7. Page 12 line 20 - clarify that groups 1 and 3 watch the video pre and post op

8. Clarify whether the same physician provides the consult in all 4 groups. If so, this is a source of bias as they can knowingly/unknowingy alter what they do for each gp given they know the allocation of each person. It looks like you refer to this as experimenter effect as a good thing but it is a source of bias. Please explain why this in not an issue. On page 13, line 10. It looks like maybe the physicians are blind to allocation, but it is not clear how they remain blind eg who gives the patient a video?

9. Page 14, line 3 - "The study investigated..: change tense or are you referring to a prior study?

10. Page 14 - the exploratory measures seem very much an unrelated substudy , at least to the question of expectations as it is written. It may be more palatable to say that better education pre-op may lessen stress therefore inflammation etc thus we can see the relevance (as opposed to opportunistic study as it reads now).

11. Page 14 - statistical analysis – are all the outcomes continuous? If not, what is the plan for categorial outcomes?

12. It is unclear what the plan is for analysing expectations. For example, is it a modifier or an outcome in itself?

13. Page 15 – sample size. Can you provide a reference for your previous work?

14. Page 16 – should include plan for managing missing data. Also what is the plan if person only receives half the intervention? Are they protocol violation? Is there a plan for analysis for 'as treated' or 'per protocol'?

15. Again relating to missing data, how are diarized pain scores being collated (eg how many daily scores and is then average taken?)

16. Are you monitoring complications? These could modify the outcomes measured.

17. How are outcomes being collected? By mail or in person and by whom?

Table 1 - define T1 etc in legend

VERSION 1 - AUTHOR RESPONSE

Reviewer: 1
Prof. Justine Naylor, Ingham Institute for Applied Medical Research, University of New South Wales
Comments to the Author:
The manuscript describes a study protocol re: a RCT to improve patient expectations pre-op in order to improve outcomes post-op.
The design is robust and the study fills a research gap.

Response: Thank you very much for your thorough and constructive review. We are grateful for your positive assessment of the study design and its clinical relevance.

TITLE
There is an assumption that the intervention will optimize expectations. In Methods, ideally you

would state which direction expectations scores will go in to reflect optimization.

Response: Thank you for the suggestion. We have revised the title to clearly indicate the research objective, study design and setting, as well as the fact that this is a study protocol. Additionally, we have clarified in the abstract and methods section that the interventions aim to enhance positive treatment expectations as the intended direction of change.

ABSTRACT

1. LINE 15 - It is not clear why these things are crucial. Rising numbers does not in itself make these things crucial. Re-phrase

Response: Thank you for your helpful comment. We have revised the sentence to clarify that the need for optimised perioperative care and pain management is based on the significant overall impact of osteoarthritis (OA) and the regular use of total hip arthroplasty (THA), rather than increasing case numbers alone.

2. Consider changing abbreviation for DOC group or change it to mean "augmented doctor conversation" to make abbreviation make sense?

Response: Thank you for the suggestion. To improve clarity and ensure consistency with the intervention content, we have revised the abbreviation from **DOC** to **aDOC** (augmented doctor conversation) throughout the manuscript.

BACKGROUND

- LINE 17, page 5 – What considerable risk? Is it not mentioned prior to this.

Response: Thank you for highlighting this. We have revised the paragraph to clarify that persistent postoperative pain is a documented risk following THA, affecting a relevant proportion of patients. We now cite several studies to support this, including a systematic review by Beswick et al. (2012), which reports that a considerable number of patients continue to experience long-term pain following THA. Additionally, we included recent studies demonstrating that while THA significantly improves pain, function, and quality of life, a subgroup of patients reports persistent postoperative limitations (Martinez-Cano et al., 2016; Vickery et al., 2025). These references further support the need to improve perioperative care and pain management in this population.

- Suggest line 58, page 5, is not a new paragraph

Response: Thank you for the suggestion. To enhance continuity and improve the overall readability of the background section, we have removed the paragraph break and integrated the sentence into the preceding text.

- Page 6 line 19 - be consistent with terms hip replacement or hip arthroplasty? (and page 16, line 51)

Response: Thank you for pointing this out. We have revised the manuscript to consistently use the term hip arthroplasty.

- The review of what is known/has been done re: THA is a little light. Could be bolstered. Much of what is cited relates to theory of expectations/learnings et from elsewhere with little reference to what is known specifically in THA. A quick search of literature identified these papers that I don't think have been cited. It is clear from below list, not much has been done in this space, thus the study is warranted.

- Mooiweer Y, Roling L, Vugrin M, Ansmann L, Stevens M, Seeber GH. Influence of patients' preoperative expectations on postoperative outcomes after total knee or hip arthroplasty: a systematic review. EFORT Open Rev. 2024 Feb 1;9(2):107-118. doi: 10.1530/EOR-23-0087.

• Tilbury C, Haanstra TM, Verdegaal SHM, Nelissen RGHH, de Vet HCW, Vliet Vlieland TPM, Ostelo RW. Patients' pre-operative general and specific outcome expectations predict postoperative pain and function after total knee and total hip arthroplasties. Scand J Pain. 2018 Jul 26;18(3):457-466. doi: 10.1515/sjpain-2018-0022.
• Neuprez A, Delcour JP, Fatemi F, Gillet P, Crielaard JM, Bruyère O, Reginster JY. Patients' Expectations Impact Their Satisfaction following Total Hip or Knee Arthroplasty. PLoS One. 2016 Dec 15;11(12):e0167911. doi: 10.1371/journal.pone.0167911.
• Laferton JAC, Oeltjen L, Neubauer K, Ebert DD, Munder T. The effects of patients' expectations on surgery outcome in total hip and knee arthroplasty: a prognostic factor meta-analysis. Health Psychol Rev. 2022 Mar;16(1):50-66. doi: 10.1080/17437199.2020.1854051. Epub 2020 Dec 21. PMID: 33228474.
• Mancuso CA, Sculco TP, Salvati EA. Patients with poor preoperative functional status have high expectations of total hip arthroplasty. J Arthroplasty 2003;18 (7):872-878.

<u>Response:</u> Thank you for this valuable suggestion. We agree that previous citations focused primarily on general theories of treatment expectations and evidence from non-orthopaedic surgical contexts. In response, we have revised the background section to include several key studies that directly examine the role of expectations in total hip and knee arthroplasty outcomes [Mooiweer et al., 2024; Tilbury et al., 2018; Neuprez et al., 2016; Laferton et al., 2022; Mancuso et al., 2003]. These references strengthen the rationale for our study and highlight the clinical relevance of expectations in the specific context of THA.

METHODS

1. Page 8, line 19-24. It is unclear what is meant by “projects” can a different term be used or explain? Central projects vs participating projects?

<u>Response:</u> Thank you for the helpful feedback. We have revised the text to make it clearer that the study is part of a larger Collaborative Research Centre (CRC), which is itself composed of several sub-projects. In this context, 'central projects' refers to overarching research components that are coordinated across all sub-projects, and the current trial is one example of a participating sub-project. The terminology has been adjusted to improve clarity.
Page 9 exclusion criteria - clarify what is meant by secondary THA (revision or had previous THA on other side?) Note- prior primary THA (on contralateral side) could be a modifier. In a related thought, assume people can’t be enrolled in the study twice Eg if come back for 2nd joint within study time frame

<u>Response:</u> Exclusion criteria comprise the revision of total hip arthroplasty (THA) procedures as well as any history of prior THA on the contralateral side. Individuals meeting either of these criteria will not be eligible for study participation. These patients are excluded to avoid potential bias arising from previous THA-related experiences—whether positive or negative— which could influence their expectations and postoperative outcomes. To avoid duplication, patients can only be enrolled once and cannot participate again if undergoing a second THA during the study period.

2. Page 9, exclusion criteria - How is opioid use determined and classified eg any use or daily use or dose dependent?

<u>Response:</u> Thank you for pointing this out. Opioid use is assessed and documented during the medical history interview based on patients’ self-reported information. The intake is evaluated by enquiring about dosage, frequency, and duration of use. The reported information is classified according to the ICD-10 criteria F11.1 (harmful use of opioids) and F11.2 (opioid dependence syndrome). Patients with F11.1 or F11.2 will be excluded, to avoid bias due to long-term neurobiological and psychological changes associated with opioid use disorder. However, it is very important to stress, that patients will not be excluded for temporary opioid intake prescribed to bridge the time interval until THA-surgery.

3. Page 9, exclusions – why is pregnancy/breast feeding an issue? Normally explain when it is unusual (would the interventions affect these or doe the assessments contraindicate these) (I note people who are pregnant/breast-feeding are not normally undergoing major

arthroplasty anyway so not usually stated)

Response: Thank you for this comment. While we agree that individuals who are pregnant or breastfeeding rarely undergo elective total hip arthroplasty, we have included this exclusion criterion as a precautionary measure. The study involves multiple repeated blood sampling, which could pose an additional physical burden during pregnancy or lactation. Importantly, the target population for this study primarily consists of individuals aged 60–80 years, making pregnancy or breastfeeding highly unlikely in practice. The criterion was included for completeness and to ensure consistency across study sites.

4. Page 10 line 3 - when pre-surgery is this visit? Clarify here as this is when randomization takes place and we need to know time from b/w randomization and intervention delivery (I note this is clarified in 2.4 but needs to be stated here instead)

Response: Thank you for the helpful comment. We agree, the timing of the randomisation in relation to the pre-surgical visit should be clarified. We have revised the text specify that the randomisation occurs at the baseline visit, which takes place approximately four days before surgery. The assigned intervention (augmented doctor conversation and/or video) is delivered on the same day, immediately after the baseline assessment.

5. Page 10, line 45 - are German THA patents really on average 60 yrs old? This seems relatively young.

Response: Thank you for your observation. The average age of patients undergoing total hip arthroplasty (THA) in Germany is indeed younger than in some other countries. According to data from the German Federal Statistical Office (Destatis) and the German Endoprosthesis Registry (EPRD), the mean age at primary THA is approximately 66 years, with substantial variability depending on comorbidities and indication (https://register.awmf.org/assets/guidelines/187-001l_S3_Indikationskriterien_Hüfttotalendoprothese_bei_Coxarthrose_2021-04.pdf?utm); Mooiweer Y, Stewart R, Dijkstra B, Oosterveld R, Stevens M, Seeber GH. Total Hip Arthroplasty: The Influence of Different Management Structures in Germany and the Netherlands on Preoperative Expectations, Self-Efficacy, and Postoperative Satisfaction. Cureus. 2025. We have adjusted the text in the manuscript to reflect this more accurately.

6. Page 12 line 20 - clarify that groups 1 and 3 watch the video pre and post op

Response: Thank you for your comment. We have clarified in the manuscript that participants in groups 1 and 3 (Video and aDOC+Video) will view the video intervention twice—once during the preoperative visit and again on postoperative day 2.

7. Clarify whether the same physician provides the consult in all 4 groups. If so, this is a source of bias as they can knowingly/unknowingly alter what they do for each gp given they know the allocation of each person. It looks like you refer to this as experimenter effect as a good thing but it is a source of bias. Please explain why this in not an issue. On page 13, line 10. It looks like maybe the physicians are blind to allocation, but it is not clear how they remain blind eg who gives the patient a video?

Response: Thank you for this important point! We agree that unblinded physician interactions can introduce bias. Physicians are not involved in group allocation and are kept blinded whenever possible. However, for the augmented physician communication group (aDOC and aDOC+Video), the physicians conducting the structured, expectation-enhancing consultation must be aware of their role, as this is inherent to the intervention. These participants are scheduled with trained physicians who deliver the intervention according to a predefined script. Participants in the TAU and Video-only groups receive standard preoperative consultations from other clinicians uninvolved in the study. To prevent contamination and minimise bias, the consulting physicians are not informed about the specific group assignments of participants outside of the aDOC groups. The video intervention is administered separately by study staff after the physician consultation, ensuring that the consulting physicians do not know whether a participant has received the video intervention. We have revised the manuscript to make these

procedures more transparent and to address the risk of experimenter bias.

8. Page 14, line 3 - "The study investigated..: change tense or are you referring to a prior study?

Response: Thank you for your comment. The sentence referred to the current study; therefore, we have adjusted the verb tense to the future form ("will investigate") to accurately reflect that this is a study protocol outlining planned procedures that have not yet been completed.

9. Page 14 - the exploratory measures seem very much an unrelated substudy , at least to the question of expectations as it is written. It may be more palatable to say that better education pre-op may lessen stress therefore inflammation etc thus we can see the relevance (as opposed to opportunistic study as it reads now).

Response: Thank you for this helpful suggestion. We agree that the link between expectation optimisation and inflammatory processes needed to be explained more clearly. We have therefore revised the manuscript to explicitly state that enhanced preoperative communication and expectation management could potentially reduce psychological stress and, consequently, reduce systemic inflammation. This provides a rationale for investigating the relationship between treatment expectations and inflammatory responses as a secondary objective within the current study framework.

10. Page 14 - statistical analysis – are all the outcomes continuous? If not, what is the plan for categorial outcomes?

Response: Thank you for this important point. While most of the primary and secondary outcomes in our study are continuous (e.g. pain intensity, functional scores, self-efficacy, inflammatory markers), a small number are categorical, such as the presence of complications or medication use. We have revised the statistical analysis section to clarify that categorical outcomes will be analysed using appropriate methods such as chi-square tests or logistic regression, depending on the data type and distribution.

11. It is unclear what the plan is for analysing expectations. For example, is it a modifier or an outcome in itself?

Response: Thank you for your comment. Expectations will be analysed as both an outcome and a potential moderator of the effects of treatment on postoperative outcomes such as pain and recovery, by assessing changes from baseline to follow-up. This dual approach reflects the current evidence that suggests expectations can influence treatment response and clinical outcomes. This has been clarified in the revised manuscript.

12. Page 15 – sample size. Can you provide a reference for your previous work?

Response: Thank you for the suggestion. We have added references to our previous work on similar expectation-based interventions to support the sample size justification.

13. Page 16 – should include plan for managing missing data. Also what is the plan if person only receives half the intervention? Are they protocol violation? Is there a plan for analysis for 'as treated' or 'per protocol'?

Response: Thank you for this important point. We have added a section describing our approach to missing data and protocol adherence. Missing data will be handled using multiple imputation where appropriate. In cases of extensive missing data (e.g. absence of primary outcome at all timepoints, "half of the intervention"), participants will be excluded from the respective analysis. In addition to the primary intention-to-treat (ITT) analysis, a per- protocol sensitivity analysis will be performed, excluding participants with major protocol deviations

(e.g. receiving only part of the assigned intervention). These changes have been incorporated into the statistical analysis section of the manuscript.

14. Again, relating to missing data, how are diarized pain scores being collated (eg how many daily scores and is then average taken?)

Response: Thank you. Pain diary entries are collected during the inpatient stay, covering approximately the first 3–4 postoperative days. These daily ratings will be calculated to generate a mean early postoperative pain score for use in the analysis. We have clarified this in the manuscript.

15. Are you monitoring complications? These could modify the outcomes measured.

Response: Thank you for this important suggestion. Yes, postoperative complications will be recorded from medical records and categorised according to severity. These data will be considered in exploratory analyses to assess their potential influence on patient-reported outcomes.

16. How are outcomes being collected? By mail or in person and by whom?

Response: Outcome assessments during the inpatient stay are conducted in person by study staff. Follow-up assessments are conducted either in person during scheduled hospital visits or by mail, depending on the time point and patient availability. Data collection is carried out by study staff who are not involved in delivering the intervention.

Table 1 - define T1 etc in legend

Response: Thank you for your comment. Your comment encourages us to reconsider the naming of the time points. In favour of greater clarity, we have chosen new naming terms (see Table 1). We have added definitions of all time points (e.g., T1, T2) to the legend of Table 1.

Reviewer: 1 competing interests.: I have just completed a QI study looking at the effect of exposing people to a dose of reality pre arthroplasty on expectations. We have also designed a related longitudinal study.

Response: Thank you for sharing your related work. We greatly value your expertise in the field and the valuable feedback you have provided. Your insights, particularly with regard to the impact of expectations on arthroplasty outcomes, have strengthened the theoretical basis and methodological clarity of our study. It is encouraging to see that similar research efforts are underway, highlighting the growing relevance of this topic in orthopaedic care.

VERSION 2 - REVIEW

**Reviewer**	**1**
**Name**	**Naylor, Justine**
**Affiliation**	**Ingham Institute for Applied Medical Research, Whitlam Orthopaedic Research Centre**
**Date**	**19-Oct-2025**
**COI**	

The authors have revised the manuscript comprehensively according to feedback

VERSION 2 - AUTHOR RESPONSE

Reviewer: 1
Prof. Justine Naylor, Ingham Institute for Applied Medical Research, University of New South Wales
Comments to the Author:
The authors have revised the manuscript comprehensively according to feedback

Response: We appreciate your acknowledgement. We have carefully considered all the comments from the reviewers and editors, and revised the manuscript accordingly to improve its clarity, structure and consistency.

Reviewer: 1 competing interests: Recently completed own expectations study in this field.

Response: Thank you for disclosing this information. We value your expertise in the field and value your constructive feedback throughout the review process.
